# Eribulin inhibits growth of cutaneous squamous cell carcinoma cell lines and a novel patient-derived xenograft

**DOI:** 10.1038/s41598-023-35811-3

**Published:** 2023-05-27

**Authors:** Che-Yuan Hsu, Teruki Yanagi, Takuya Maeda, Hiroshi Nishihara, Kodai Miyamoto, Shinya Kitamura, Keiko Tokuchi, Hideyuki Ujiie

**Affiliations:** 1 Department of Dermatology, Faculty of Medicine and Graduate School of Medicine, Hokkaido University, N15 W7, Kita-Ku, Sapporo, 060-8638 Japan; 2 Genomics Unit, Keio Cancer Center, Keio University School of Medicine, Tokyo, Japan

**Keywords:** Cancer, Genetics

## Abstract

Advanced cutaneous squamous cell carcinoma (cSCC) is treated with chemotherapy and/or radiotherapy, but these typically fail to achieve satisfactory clinical outcomes. There have been no preclinical studies to evaluate the effectiveness of eribulin against cSCC. Here, we examine the effects of eribulin using cSCC cell lines and a novel cSCC patient-derived xenograft (PDX) model. In the cSCC cell lines (A431 and DJM-1 cells), eribulin was found to inhibit tumor cell proliferation in vitro as assessed by cell ATP levels. DNA content analysis by fluorescence-activated cell sorting (FACS) showed that eribulin induced G2/M cell cycle arrest and apoptosis. In xenograft models of cSCC cell lines, the administration of eribulin suppressed tumor growth in vivo. We also developed a cSCC patient-derived xenograft (PDX) which reproduces the histological and genetic characteristics of a primary tumor. Pathogenic mutations in *TP53* and *ARID2* were detected in the patient’s metastatic tumor and in the PDX tumor. The cSCC-PDX responded well to the administration of eribulin and cisplatin. In conclusion, the present study shows the promising antineoplastic effects of eribulin in cSCC. Also, we established a novel cSCC-PDX model that preserves the patient’s tumor. This PDX could assist researchers who are exploring innovative therapies for cSCC.

## Introduction

Cutaneous squamous cell carcinoma (cSCC) accounts for 20% of keratinocyte malignancies and more than 70% of all non-melanoma skin cancer deaths. The majority of primary cSCC can be effectively cured with surgical excision, but a small percentage of patients have characteristics that are associated with a high likelihood of local recurrence, distal metastasis, and death^1^. Since no accepted systemic therapy or standard care for advanced cSCC has been developed, there is a need for new forms of treatment. Multi-drug chemotherapy appears to have higher efficiency than mono-drug regimens, but adverse events are a great concern. Available treatment choices are chemotherapy (cisplatin ± 5-fluorouracil) with concurrent radiotherapy, targeted therapy with EGFR inhibitors (e.g. cetuximab), and anti-PD-1 antibodies (cemiplimab, pembrolizumab, nivolumab)^2^. To investigate effective therapies that have fewer side effects than current ones, it is essential to more precisely characterize the molecular mechanisms and pharmacodynamics related to cutaneous SCC pathogenesis and to discover innovative drug targets.

Eribulin is a non-taxane synthetic analog of halichondrin B that was originally found in the marine sponge *Halichondria okadai*. It was proven effective in clinical trials as an antineoplastic agent for metastatic breast cancer^3,4^ and unresectable liposarcoma^5^. Eribulin has a significant inhibitory effect on tumors in vitro at the sub-nanomolar (nM) level against various human cancer cell lines. Also, eribulin displays prominent in vivo anti-carcinogenicity in human xenografts^6^. In addition to the anti-carcinogenicity that eribulin achieves by inhibiting microtubule dynamics, the agent plays a role in mechanisms such as tumor microenvironment alteration^7^, the reversion of epithelial mesenchymal transition (EMT)^8,9^, the inhibition of TGF-β signaling^10^, vascular remodeling^11^, the elimination of tumor hypoxia^12^, the inhibition of tumor migration invasion and metastasis^13^, and the inhibition of telomerase reverse transcriptase RNA-dependent RNA polymerase (TERT-RdRP)^14^. Previous published results from our lab have shown eribulin to be effective against xenografted tumors of extramammary Paget’s disease (EMPD)^15^. Even though there have been no preliminary data on the effectiveness of eribulin against cSCC, our previous results brought to our attention the possibility of using eribulin as a therapy for cSCC.

In the last few years, the advantages and applications of patient-derived xenograft (PDX) models have been reported for numerous cancer types^16,17^. PDX models can maintain their tumor prototypes and can be used for preclinical treatment research on certain cancers. These models have demonstrated that they can help predict the clinical prognosis of patients and are aimed at anti-cancer drug screening and assessment, biomarker recognition, and customized disease-treatment strategies^16^.

Herein, we assess the anti-proliferative activity of eribulin by using cSCC cell lines and a novel cSCC-PDX model.

## Results

### Eribulin suppresses the tumor growth of cSCC cell lines in vitro

**Figure 1:**
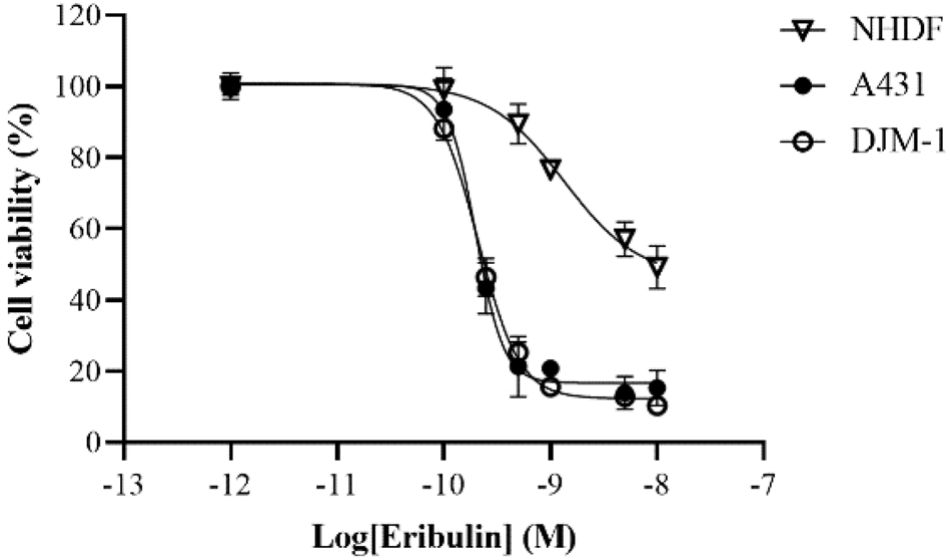
Eribulin suppresses tumor growth in cSCC cell lines in vitro. The sensitivity to eribulin of two cSCC cells (A431 and DJM-1) and of normal human dermal fibroblasts (NHDFs) was determined by ATP assay. 2000 cells per well were seeded in 96-well plates for 24 h. Following that, the cells were treated with eribulin at various concentrations (0.001, 0.1, 0.25, 0.5, 1, 5, 10 nM). After 72 h, cell viability was assessed using CellTiter-Glo^®^ 2.0 assay (Promega). The x-axis indicates the log value of eribulin concentration; the y-axis indicates the relative cell viability (normalized by no treatment). Data are presented as mean ± SD from triplicate experiments.

Based on the well-known anti‑tubulin activity of eribulin^18,19^, we first examined the half-maximal inhibitory concentration (IC_50_) of eribulin against two cSCC cells (A431 and DJM-1) and against normal human dermal fibroblasts (NHDFs) (Fig. 1). One day after seeding, eribulin was added to each cell line in serially diluted concentrations (0.001, 0.1, 0.25, 0.5, 1, 5, 10 nM) and the cultures were incubated for 3 days. There was no remarkable difference in IC_50_ concentration between the two cSCC cell lines (A431: 0.20 nM; DJM-1: 0.21 nM). The IC_50_ was markedly higher for NHDFs (1.34 nM) than for other cells. Meanwhile, the keratinocyte-derived cSCC cell lines were approximately seven times more sensitive to eribulin than NHDFs were.

### Eribulin induces cell cycle arrest and cell death in cSCC cell lines

**Figure 2:**
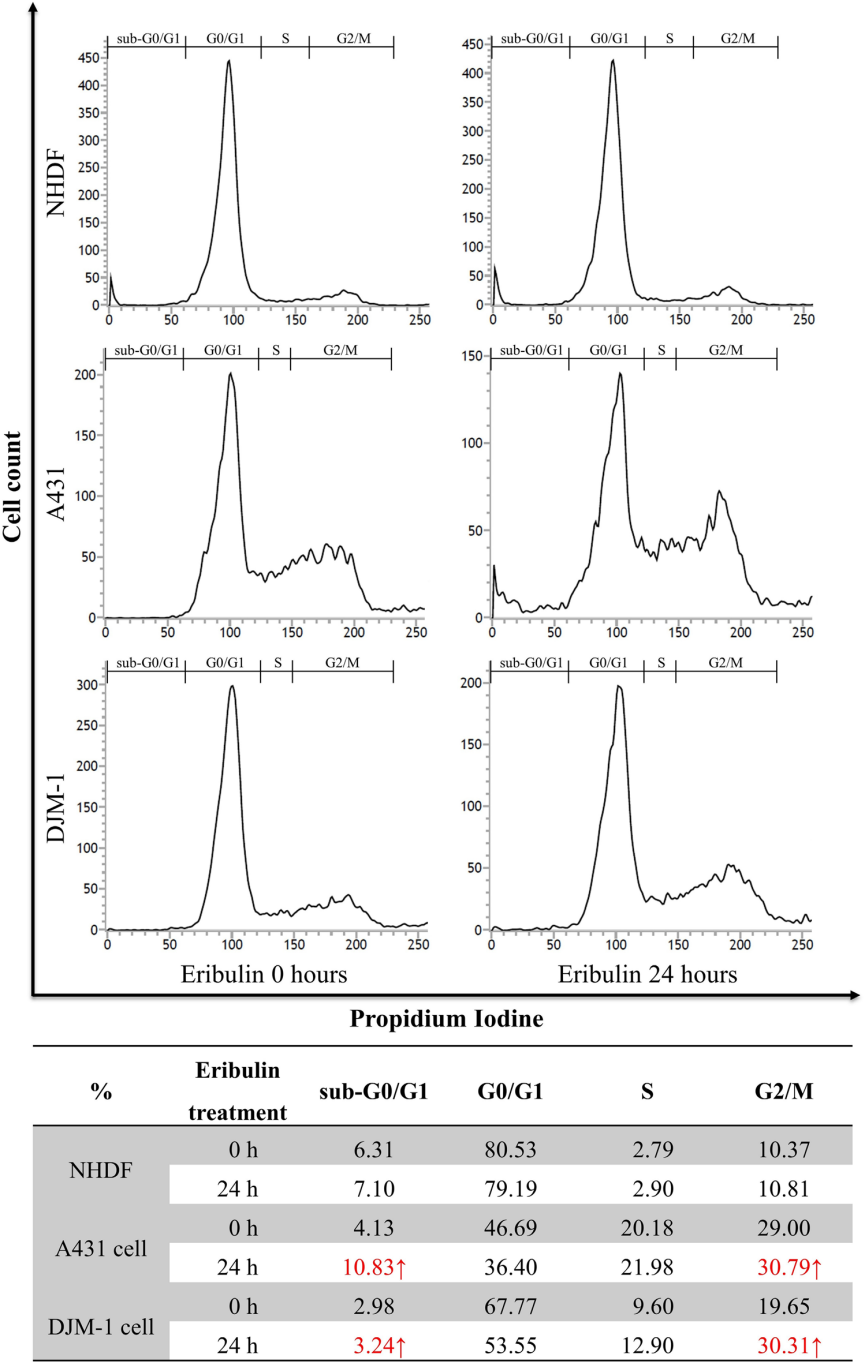
Eribulin induces cell cycle arrest at the G2/M phase in cSCC cell lines. cSCC cells (A431 and DJM-1) and NHDF were cultured with or without 0.5 nM of eribulin for 24 h. Cells were fixed in 70% ethanol and treated with RNase A. Cells were stained with DNA-binding fluorochrome propidium iodide. DNA contents were determined by fluorescence-activated cell sorting (FACS) analysis. The x-axis indicates propidium iodide fluorescence; the y-axis indicates cell counts. Data are representative of duplicate experiments.

Since the cSCC cells (A431 and DJM-1) showed high sensitivity to eribulin in vitro, DNA content analysis using flow cytometry was performed to investigate the effect of eribulin on cell cycle progression. At 24 h after the administration of eribulin, the subgroups of A431 and DJM-1 cells that were in the G2/M and sub-G0/G1 phases were increased. Conversely, the subgroups of A431 and DJM-1 cells that were observed in the G0/G1 phase were significantly decreased. NHDF cells presented no significant impact on cell cycle phases (Fig. 2). Furthermore, an increased ratio of early apoptosis was found in A431 cells, and late apoptosis in DJM-1 cells (Supplemental Fig. S1). This result revealed that eribulin induces apoptosis in A431 cells and induces cell death in DJM-1 cells. This finding indicates that the eribulin induced G2/M mitotic block and cell death, as previously reported^6,18,19^.

### Administration of eribulin inhibits tumor growth in vivo

**Figure 3:**
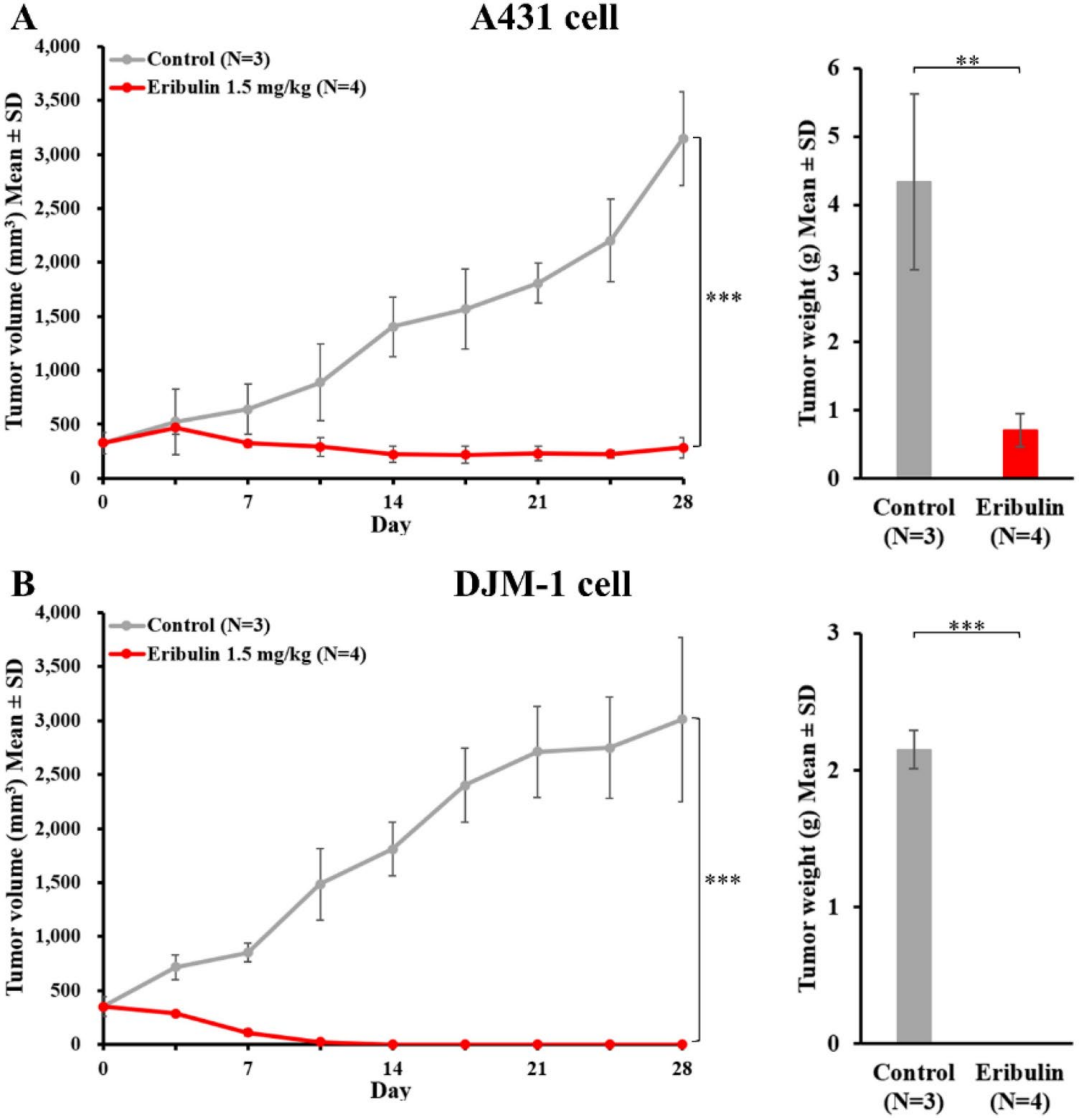
Administration of eribulin inhibits tumor growth in vivo. A431 (**A**) and DJM-1 (**B**) cells (5 × 10^6^) were injected subcutaneously into the bilateral flanks of *nu/nu* mice. When the tumors reached 8 mm in diameter, eribulin was administrated via the tail vein once a week. Tumor volumes were measured twice per week and were estimated based on the formula: (long axis × short axis^2^)/2. The tumors were extracted and measured at 28 days after treatment initiation. The results are shown as mean ± SD. **P < 0.01, ***P < 0.001.

To extend the studies and elucidate the effect of eribulin in vivo, we subcutaneously xenografted cSCC cells (A431 and DJM-1) into immunodeficient nude mice. Compatible with in vitro experimental evidence, both the A431 and DJM-1 xenograft tumors showed reduced growth curves under eribulin treatment (1.5 mg/kg injected intravenously once a week). The weights of extracted tumors in the eribulin-treated group were significantly lower than those in the control group (Fig. 3A and B). Taken together, the above data reveal that the systemic administration of eribulin suppresses the tumor growth of cSCC in vivo.

### Establishment of a cSCC-PDX mouse model harboring *TP53* and *ARID2* mutations

**Figure 4:**
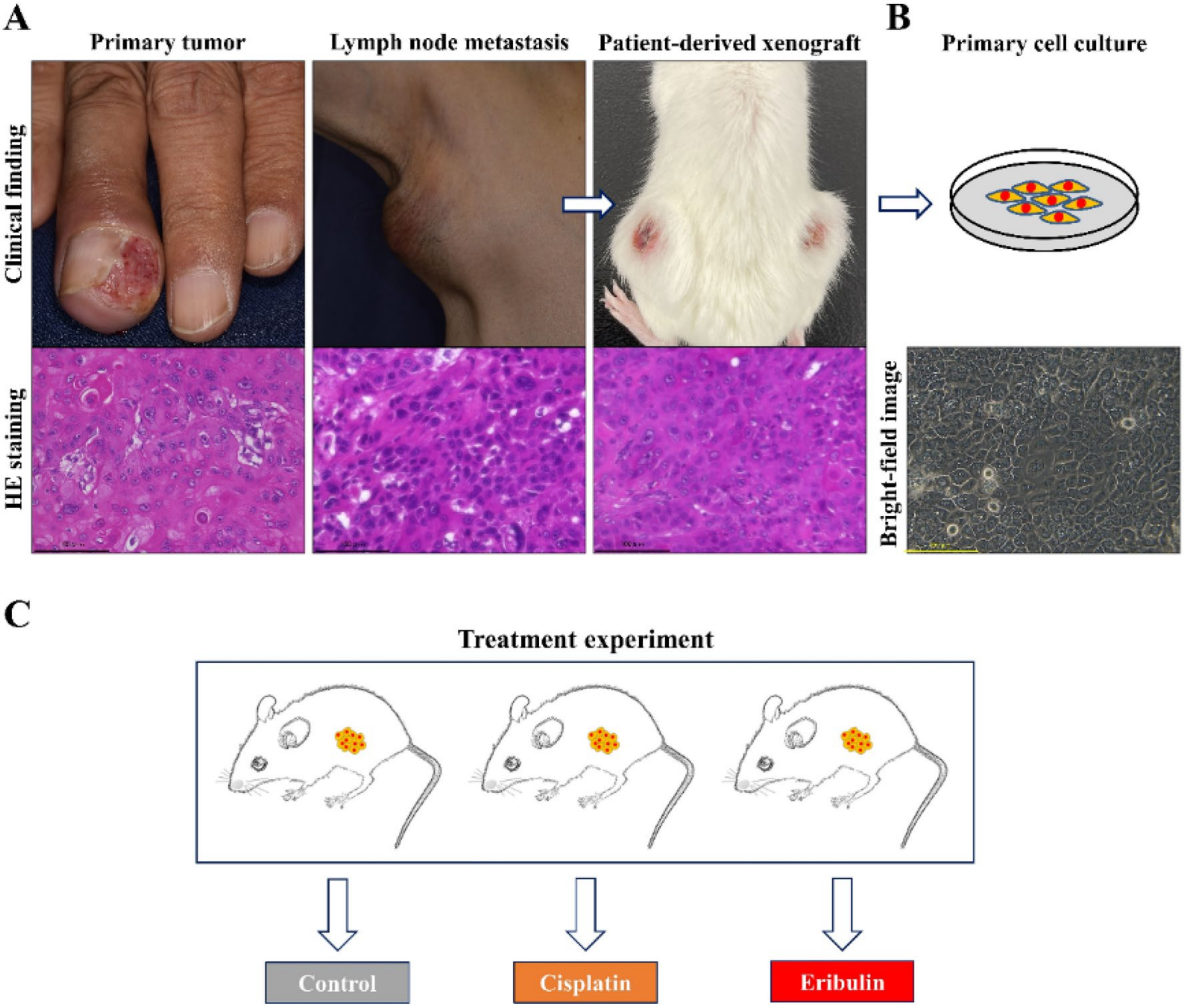
Schematic of the establishment of the cSCC-PDX mouse model. (**A**) The patient’s primary skin lesion was located on the ring finger of the right hand (upper left). Lymph node metastasis was observed 2 years after the first surgery (upper middle). The extracted tumor from the lymph node metastasis was transplanted into NOD/SCID mice (G0, upper right). The enlarged xenografted tumors were transplanted to further generations for primary cell cultures (**B**) and for treatment study (**C**). In HE staining and bright-field images, the cSCC-PDX tissue exhibits similar morphology to that of the patient’s tissue (metastatic lymph node). (**C**) In treatment experiments, tumor-bearing NOD/SCID mice were randomized into a control group, a cisplatin group (5 mg/kg/week), and an eribulin group (1.5 mg/kg/day).

Few available cSCC-PDX models have been reported^20^; thus, we tried to develop a mouse model for assessing the efficacy of eribulin (Fig. 4)^21^. Surgically excised tissue was transplanted into the flanks of nonobese diabetic/severe combined immunodeficient (NOD/SCID) mice. The transplanted cSCC tumor arose from the host and enlarged into a subcutaneous indurated nodule more than 10 mm in diameter over a period of 4 months (generation 0: G0). Once the tumor volume exceeded 1000 mm^3^, the cSCC-PDX tumors were transplanted into the next generation of NOD/SCID mice. The patient specimen and xenograft tissue were histologically reviewed by hematoxylin and eosin (HE) staining. The cSCC-PDX tissue manifested identical morphology to that of the patient tissue (primary tumor and lymph node metastasis) (Fig. 4A). Also, we established primary culture cells from the third generation of cSCC-PDX tumors, in which the cultured cells were epithelioid (Fig. 4A). To determine whether the patient’s tissues and the cSCC-PDX tumors had consistent genetic similarities, we conducted gene mutation analysis of cancer-associated genes (Supplemental Table S1). Several pathogenic gene mutations, including *TP53* R175Pfs*2, *ARID2* T1167Lfs*6, *BRCA1* S1563C, and *BRCA1* E1562Q, were observed to be shared by the patient’s cSCC tumor and the cSCC-PDX tumors. The xenograft passage had good concordance with the corresponding tumor tissue of the patient (Supplemental Table S2). In the patient’s lymph node, the variant allele frequency (VAF) for *TP53* R175Pfs*2 and *ARID2* T1167Lfs*6 was 97.2% and 58.3%, respectively. As a result of normal allele loss and the ratio of normal cells diminished in the cSCC-PDX (G1) tumor, the VAF of both mutations was raised to just under 100%. Additionally, there were many more genetic alternations in xenografts due to mutations of several tumor suppressors, including *ARID2*^22^.

### Eribulin suppresses tumor growth in the novel cSCC-PDX mouse model

**Figure 5:**
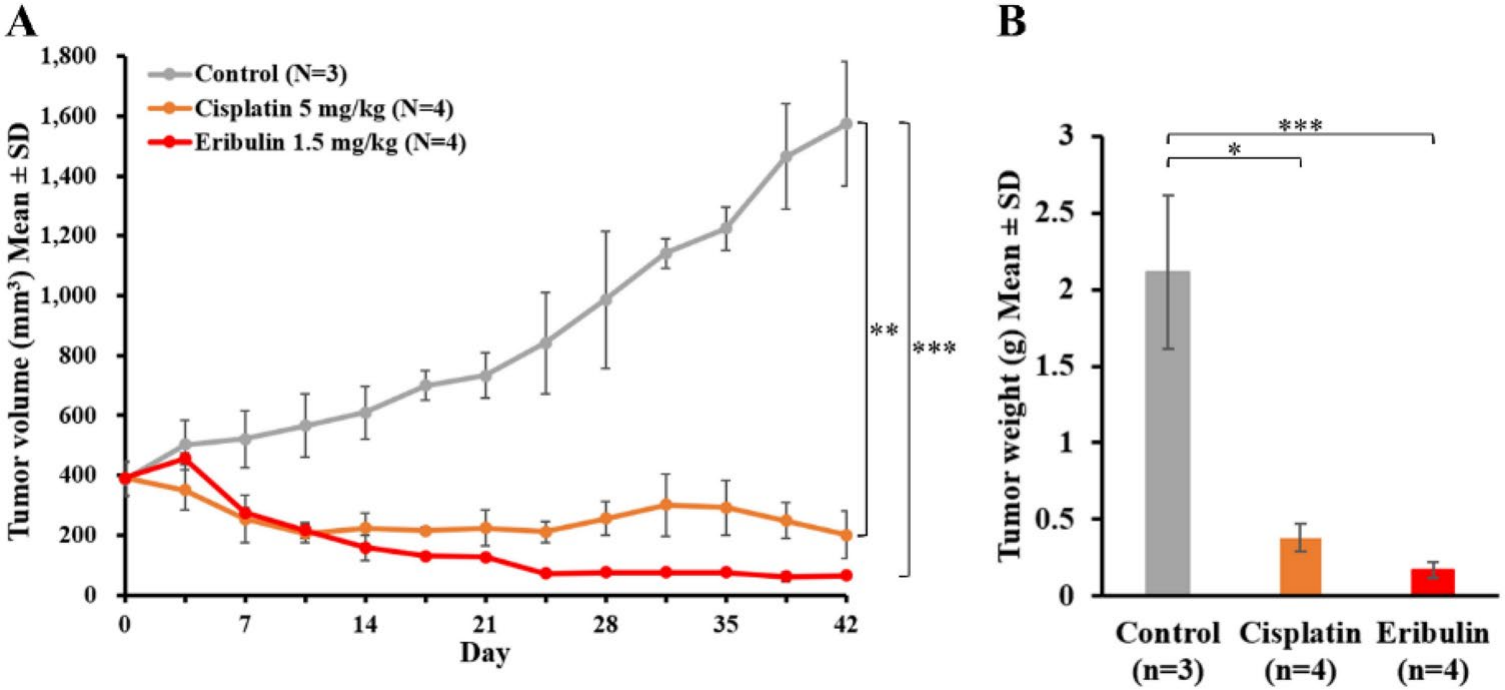
Cytotoxic agents including eribulin suppress tumor growth in the novel cSCC-PDX mouse model. Tumor-bearing NOD/SCID mice were randomly assigned to a control group (grey line), a cisplatin group (orange line), or an eribulin group (red line). Cisplatin (n = 4, 5 mg/kg) was administered intraperitoneally once per week. Eribulin (n = 4, 1.5 mg/kg) was administered intravenously once per week. (**A**) Tumor volumes were measured twice per week. (**B**) The tumors were extracted and measured at 42 days after treatment initiation. The results are shown as mean ± SD. *P < 0.05, **P < 0.01, ***P < 0.001.

**Figure 6:**
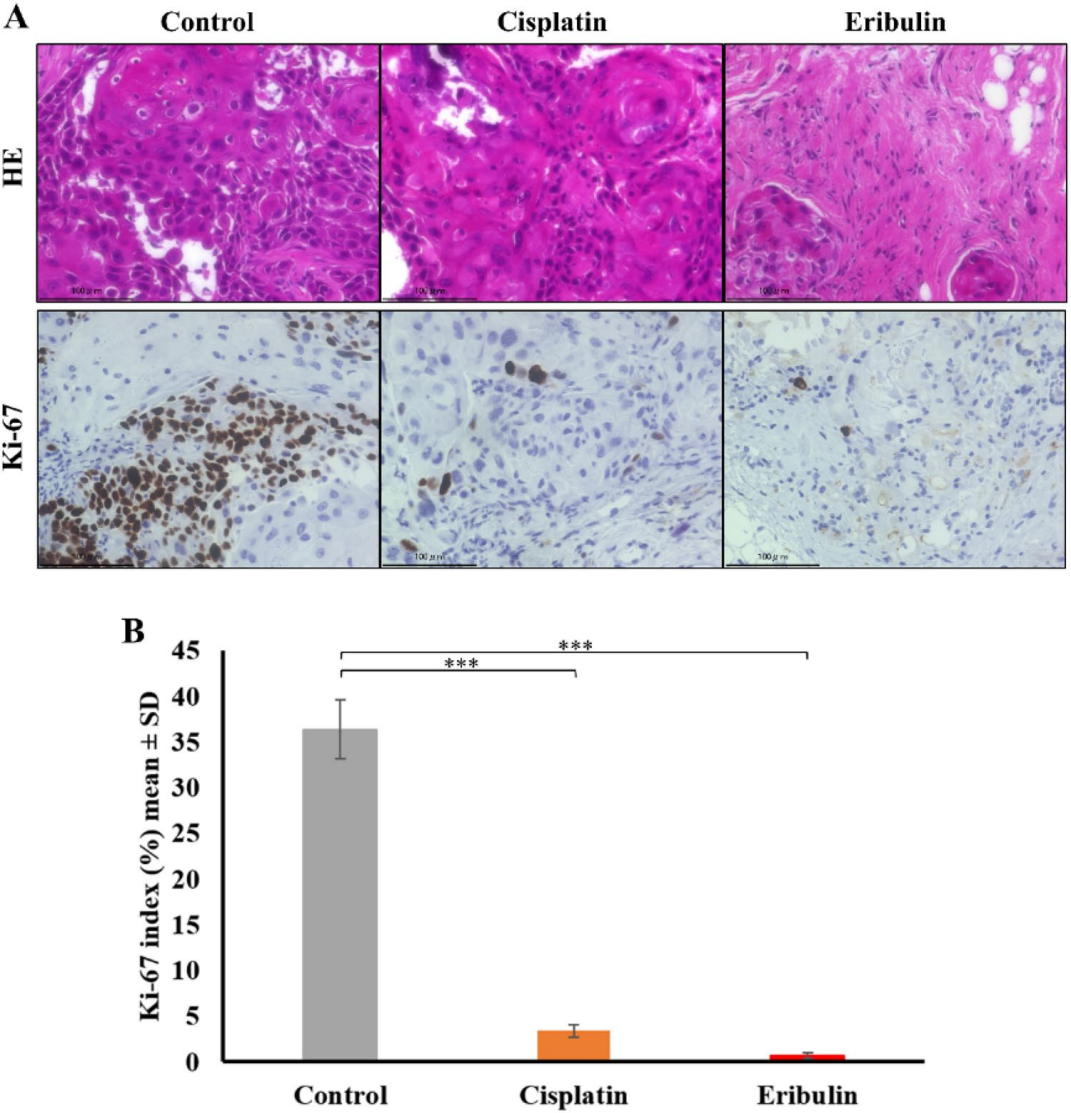
Ki-67 staining indicates that anti-neoplastic agents are effective against the cSCC-PDX tumors. To assess the therapeutic response, treated tumors were extracted at day 42 for all groups (the control, cisplatin, and eribulin groups). (**A**) The panel shows representative images of HE and Ki-67 staining of control and treated SCC-PDX tumors. (**B**) The bar chart indicates the Ki-67 index (the Ki-67-positive cell ratio (%)). The results are shown as mean ± SD. ***P < 0.001.

To date, no preclinical studies on cSCC treated with eribulin have been published. We conducted treatment experiments to study whether the novel cSCC-PDX responds to chemotherapy (Fig. 5)^23–25^. Regarding cytotoxic chemotherapies, the xenografted model responded well to cisplatin, which has been reported to be the standard medication^24,25^. Furthermore, we administrated eribulin monotherapy, which has proven effective as a second-line treatment for metastatic breast cancer^26^. As observed in other preclinical models^15^, eribulin administration (1.5 mg/kg/week) successfully suppressed tumor growth in the cSCC-PDX, and no progression was observed for 2 weeks. The eribulin-treated groups showed effects as great as those of the cisplatin-treated group. The data of treatment experiments were additionally validated by Ki-67 staining (Fig. 6). In immunohistochemical analysis, the Ki-67 index score was significantly lower in the eribulin-treated cSCC-PDX tumors than in the control tumors.

## Discussion

Cytotoxic agents for advanced cSCC include platinum-based drugs (cisplatin or carboplatin), 5-fluorouracil, bleomycin, methotrexate, doxorubicin, taxanes, capecitabine, and gemcitabine. These have all proven to have insufficient clinical efficacy, and patients have shown several adverse effects^27–29^. There have been no preclinical studies on the effectiveness of eribulin against cSCC. In this study, we presented data that demonstrate the significant anti-tumor effects of eribulin against cSCC in vitro and in vivo, suggesting that eribulin could be a novel candidate drug for cSCC treatment.

Previous studies revealed that eribulin suppresses the tumor growth of certain human cancers, including breast, lung, and ovarian cancers, in vitro (IC_50_ = 0.09 – 9.5 nM)^30,31^. In our study, cSCC cell lines (A431, DJM-1) showed relatively high sensitivity (IC_50_ = 0.20 and 0.21 nM) to eribulin, which we have not seen reported before. In cSCC cell lines with a high proliferative rate, a higher proportion of cells abnormally enter the G2/M phase of the cell cycle and cause an irreversible mitotic block. That is why cSCC cell lines are sensitive to eribulin. Even though the growth of cSCC cell lines was inhibited at the sub-nM level of drug administration, the eribulin showed no cytotoxic effects against normal human dermal fibroblasts (NHDFs) up to 1 nM, suggesting that eribulin has high potency with a wide effective range (at sub-nM concentration)^31^. Reportedly, when eribulin was administered to the xenografted mice at a dose of 1 mg/kg, the peak blood concentration exceeded 100 nM, followed by a steady low-concentration phase at around 10 nM for 1 week in xenograft tumors^32^. The present study showed the IC_50_ of eribulin in cSCC cell lines to be at the sub-nM level, suggesting that eribulin has a great anticancer activity. Eribulin blocks microtubule assembly by attaching to a distinctive binding site on tubulin that differs from the sites for other known categories of tubulin-directed drugs^33^. This unique mechanism may explain why eribulin has non-cross-resistant anti-neoplastic effects after the cessation of taxane treatment^34,35^. Regarding inhibiting mitotic spindle alignment, eribulin administration results in cell cycle arrest at the G2/M phase, leading to cell apoptosis^33^. This phenomenon was also observed in our experiments using flow cytometry analysis and immunohistochemistry.

**Figure 7:**
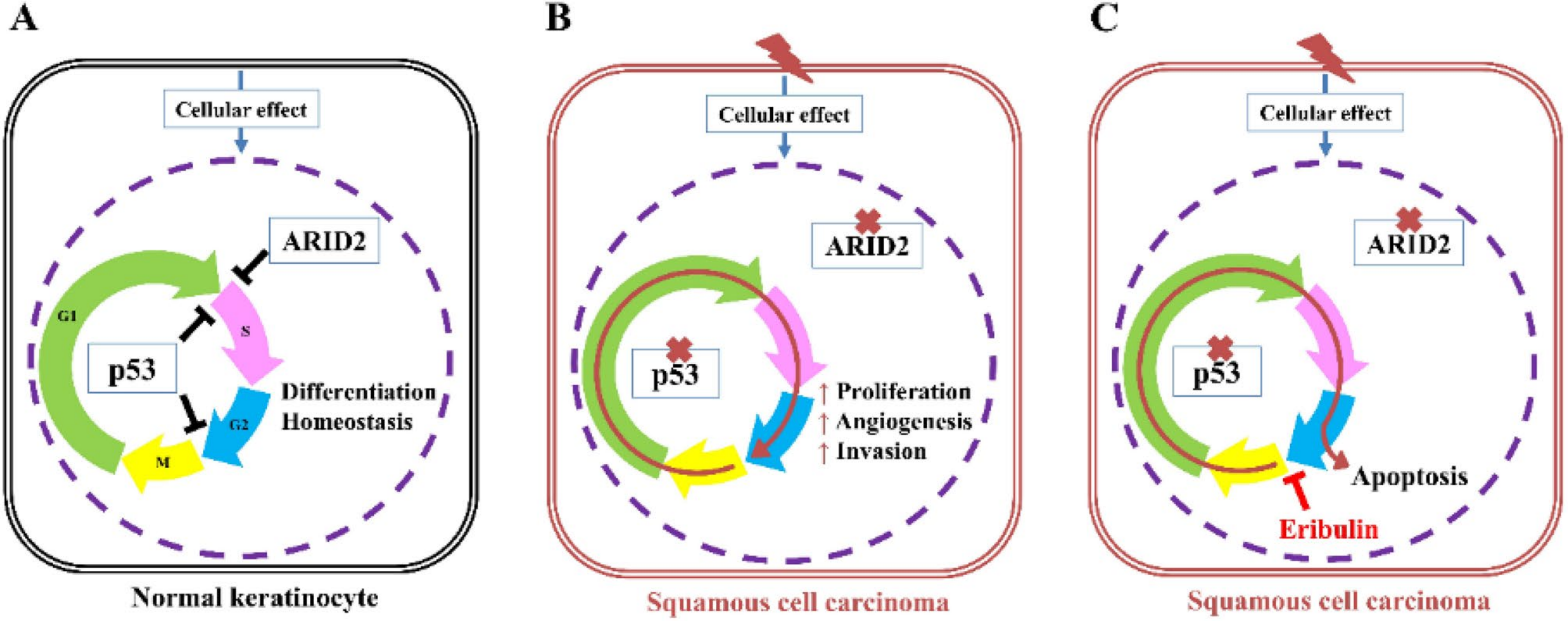
Schematic of the anti-tumor effect of eribulin on the cSCC-PDX. (**A**) In normal keratinocytes, p53 controls the G1/S and G2/M cell cycle checkpoints, and ARID2 regulates the G1/S phase transition. (**B**,**C**) In the cSCC-PDX, loss of function in p53 and ARID2 leads to the disruption of cell cycle checkpoint controls, which promotes cancer development. Eribulin induces G2/M cell cycle arrest and apoptosis in cSCC cells. The tumors with a loss of tumor suppressor function (such as p53 and ARID2) are particularly sensitive to eribulin.

In the present study, we have reported a novel cSCC-PDX mouse model harboring *TP53* R175Pfs*2 and *ARID2* T1167Lfs*6 truncating mutations. The mutations could not only result in loss of function (LOF), but could also facilitate tumor progression^36,37^. Concomitant *TP53* and *ARID2* mutation have been reported in non-small cell lung carcinoma^38^, oral SCC^39^, and colorectal cancer^40^. Concerning clinical research on *TP53* mutations in cSCC, the frequency of such mutations is around 50% in primary SCC tumors, but is almost 95% in metastatic tumors^41–43^. *TP53* controls both G1/S and G2/M cell cycle checkpoints^44^. In contrast, the incidence rate of *ARID2* genetic alteration in cSCC is unknown and has not been investigated, but it is reportedly 6% for oral SCC tumors^45^. *ARID2* performs a tumor suppressor function in *TP53-*mutated oral SCC in vitro, and *ARID2* knockdown accelerates tumor growth in vivo^36^. The inhibition of ARID2 expression enhances G1/S transition related to the upregulation of cyclin D1, cyclin E1, and CDK4 and to the phosphorylation of the retinoblastoma protein (Rb) in hepatocellular carcinoma (HCC)^46^. In several studies, *ARID2* mutations and/or lower protein expression of ARID2 have been reported to correspond to poor patient prognosis in hepatocellular carcinoma, intrahepatic cholangiocarcinoma, breast cancer, oral SCC, and lung cancer^47–51^. Based on our data, eribulin demonstrated high effectiveness against a cSCC-PDX tumor harboring *TP53* and *ARID2* mutations. Without these two tumor suppressor genes, the surveillance mechanisms at the G1/S and G2/M checkpoints fail to regulate cell cycle, leading to abnormal cell growth and tumorigenesis. The oncogenic effects of these tumor suppressor genes include the initiation of DNA synthesis and enhanced cell growth, invasion, and metastasis. After the administration of eribulin, the irreversible mitotic blockade induced by eribulin leads to cancer cell apoptosis (Fig. 7).

Our results indicate that eribulin induces irreversible mitotic blockade and apoptosis, leading to tumor regression in cSCC cell lines (A431 and DJM-1) as well as in a cSCC-PDX. Regarding A431 cells, there are no *TP53* or *ARID2* mutations; instead, the A431 cells have the amplification of *EGFR*, *CCND1*, and *CCND3* and the deletion of *CDKN2A*. The DJM-1 cells had no *TP53* or *ARID2* mutations but did have gain of function in *FGFR2* and *FGFR4*, the amplification of *MYC*, and the deletion of *CDKN2A*^52^. These cSCCs and the PDX share similarities in cell-cycle dysregulation and contribute to tumorigenesis^53^. Our data suggest that eribulin could have excellent anti-neoplastic effectiveness against cSCC cells regardless of their genetic backgrounds.

In the treatment experiment, we used cisplatin or eribulin only as a single agent. In the cisplatin group, the mice did not tolerate the drug well, becoming weak and losing body weight. In the eribulin group, the mice seemed healthy for the duration of the experiment. We may consider using eribulin in combination with low-dose cisplatin or with EGFR inhibitors as multidrug regimens in the future.

Traditional patient-derived xenograft (PDX) mouse models lack normal immunity. They cannot be used to evaluate the efficacy of anti-PD-1/PD-L1 therapies. In the past few years, some researchers have developed new mouse models where human PDX grows in the presence of a human immune system^54,55^. We may utilize this concept to establish new models for assessing the effectiveness of PD-1/PD-L1 inhibitors in combination with eribulin at a future time.

In conclusion, the present study reveals the promising antineoplastic effects of eribulin. Also, we established a novel cSCC-PDX mouse model that preserves the patient’s tumors histologically and genetically. This mouse model could help researchers who are exploring innovative cSCC therapies.

## Material and methods

### Cell lines and cell culture

The human cSCC A431 cell line^56^ and normal human primary dermal fibroblasts (NHDFs) were purchased from the American Type Culture Collection (ATCC). The human cSCC DJM-1 cells were separated from human cSCC^57^. All of the cells were cultured in Dulbecco’s modified Eagle’s medium (DMEM) (Nacalai Tesque, Kyoto, Japan) supplemented with 10% fetal bovine serum (FBS) (Sigma-Aldrich, Germany). These cells were maintained in a humidified cell incubator containing 5% CO_2_ with temperature set at 37 °C. Each cell line was confirmed by short tandem repeat profiling (Promega, WI, USA) in August 2018 and was utilized in less than 6 months of unceasing passage. All cells were verified for the absence of *Mycoplasma* contamination (Venor^®^GeM Classic, Minerva Biolabs, NJ, USA).

### Cell viability assays using ATP measurement

Cells were seeded in 96-well solid white plates at a density of 2.0 × 10^3^ cells per well in 80 μL of complete medium and were cultured for 24 h. The cells were treated with various concentrations of eribulin (0.001, 0.1, 0.25, 0.5, 1, 5, 10 nM) for 3 days. An ATP assay was performed according to the manufacturer’s protocol (CellTiter-Glo^®^ 2.0 assay, Promega). 50 μL of reagent solution was added to each well, and the plates were kept in the dark for 15 min. The absorbance of luminescence was read at 578 nm in a luminometer (SpectraMax^®^ Paradigm^®^, Molecular Devices, CA, USA)^58^.

### Cell-cycle analysis

The cells were harvested, washed with phosphate-buffered saline (PBS) (Nacalai Tesque), and fixed in cold 70% ethanol for no less than 2 h. The cells were then washed and resuspended in 500 μL of assay buffer containing propidium iodide (PI) (Sigma-Aldrich) (20 μg/ml) and ribonuclease A (RNase A, Macherey–Nagel, Germany) (200 μg/ml) for 30 min at 20 °C in the dark. The DNA contents were analyzed using FACSVerse™ and FlowJo™ v10.8.1 (BD Biosciences, NJ, USA). A total of 10 000 events were conducted and examined as described previously^59^.

### In vivo experiment on the cSCC-CDX mouse model

The cell line-derived xenograft (CDX) experiments were conducted according to our previous publication^60^. BALB/cAJcl-*nu*/*nu* mice (5 weeks old, female) were purchased from CLEA Japan, Inc. The mice in the study were fed with sterile distilled water and standard chow ad libitum. They were held in a fixed-temperature (22–25 °C) environment with a 12-h light/dark cycle under specific pathogen-free conditions. The mice were obtained and housed in accordance with the Animal Resource Program guidelines. 5.0 × 10^6^ cells were suspended in 200 μL of PBS and were injected subcutaneously into bilateral flanks. Tumor size was recorded twice per week. For the eribulin treatment group (n = 4, 1.5 mg/kg, Halaven^®^, Eisai, Tokyo, Japan), when the tumors reached 8 mm in diameter (usually 5 to 10 days after xenotransplantation), eribulin was injected into the tail vein (30 μg in 150 μL PBS, equivalent to 1.5 mg/kg) and was re-injected every week. The control xenograft tumors (n = 3) were treated with 150 μL PBS. Tumor volume was calculated by an approximation formula: ((1/2) × (major axis) × (minor axis)^2^)^59^. After 4 weeks of treatment, the tumors were extracted and the weights were recorded.

### Establishing the cSCC-PDX

cSCC tissues were obtained from the lymph node metastasis of a 69-year-old Japanese male. He had been a farmer who had habitually smoked cigarettes and drunk alcohol. The patient’s primary skin lesion was located on the finger, and he underwent wide excision with amputation for treatment. The pathological report revealed focal positivity upon microscopic examination. The enlargement of ipsilateral axillary lymph nodes was observed 2 years after surgery. The patient died soon from cancer cachexia and sepsis.

The resected metastatic lymph node was divided into two sections: one was for transplantation; the other was fixed in formalin and embedded into paraffin for pathological diagnosis. A 10-mm specimen of metastatic tissue from the cSCC patient was subcutaneously transplanted with Matrigel (BD Biosciences) into the bilateral flanks of a 5-week-old female NOD/ShiJic-*scid*Jcl mouse (CLEA Japan, Tokyo, Japan). The tumor-transplanted mouse was checked twice per week and the tumor was measured by caliper. When the tumor volume reached 1000 mm^3^, the cSCC-PDX tumors were propagated to the next generation of mice. In the first two consecutive mouse-to-mouse passages, the cSCC-PDX tumors were divided into three sections: one was cut into pieces (less than 5 mm in diameter) for transplantation, another was frozen at − 80 °C for DNA extraction, and the third was fixed with 10% formalin and then embedded in paraffin for pathological analysis. Treatment experiments were performed on the first to third generations of PDX mice. Extra amounts of fresh tumor pieces were frozen in CryoStor^®^ CS10 (BioLife Solutions, WA, USA) and stored at − 80 °C^61^. The cryopreserved cSCC-PDX tumors were re-transplanted into new mice for reanimation.

### Treatment experiments on the cSCC-PDX mouse model

Tumor growth curves of the cSCC-PDX were documented by the dynamic measurement of tumor volume. The tumor volume range of 300–400 mm^3^ in the tumor-bearing NOD/ShiJic-*scid*Jcl mice was randomly assigned, and a treatment protocol initiated. Each treatment group had a minimum of n = 3 mice per condition. The control (n = 3) mice were intravenously injected with 150 μL of PBS once per week. In the cisplatin group, cisplatin (n = 4, 5 mg/kg, FUJIFILM Wako, Tokyo, Japan) was administered intraperitoneally once per week as described by Lu et al.^62^. Eribulin (n = 4) was administered intravenously once per week^63^. The tumor volume was measured twice per week by caliper, and tumor weights were measured at 42 days after treatment initiation. Tumor volume and weight were recorded in a blinded manner.

### Histopathological analyses

Formalin-fixed, paraffin-embedded tissue sections from the patient’s tumors and the xenografted tumors were cut into 4-μm sections. Hematoxylin and eosin (HE) staining and immunohistochemistry for Ki-67 (Abcam, Camb, UK) were performed to compare the histopathology of the metastatic lymph nodes and the xenografts before versus after the treatments. DAB chromogen was applied to yield a brown color^64^. For nuclear Ki-67 expression, the percentage of positive cells among at least 100 cancer cells from three randomly selected fields of vision under high magnification (× 400) was calculated.

### Gene mutation analysis

Genomic DNA was extracted from the patient’s blood and from each tissue (patient’s lymph node and PDX) using the DNA Mini Kit (QIAGEN, Germany). The concentration and purity of DNA samples were measured using a NanoDrop 2000c Spectrophotometer (Thermo Fisher, MA, USA). DNA fragment integrity was confirmed by electrophoresis using 1% agarose gel. The concentrations of DNA samples were normalized to 20 ng/μL, and the samples were stored at − 30 °C until use. Genomic testing was performed by the genomic unit of the Keio Cancer Center in Tokyo, Japan. The quality of the DNA was first determined based on the DNA integrity number (DIN) score, which was calculated using the Agilent 2000 TapeStation system (Agilent Technologies, CA, USA), and then the targeted amplicon exome sequencing for 160 cancer-related genes was performed using the MiSeq sequencing platform (Illumina, CA, USA). The 160 cancer-related genes in the comprehensive cancer panel are listed in Supplemental Table S1. The minimum amount of DNA was 50 ng, and the minimum quality for DNA was that with a DIN score over 3.1. The sequencing data were analyzed using an original bioinformatics pipeline: GenomeJack (Mitsubishi Space Software, Tokyo, Japan).

### cSCC-PDX-derived primary cell culture

The establishment of primary culture cells was conducted as previously reported^65^. Tumor tissue from the cSCC-PDX mouse (third generation) was minced and washed with PBS repeatedly. The minced tissue was directly plated onto dishes in a medium of DMEM containing 10% FBS.

### Statistical analyses

Quantitative data are shown as mean ± standard deviation (SD). To evaluate the statistical significance of the treatment groups, the Student’s t test was used to estimate the statistical significance between each category. At least three independent experiments were carried out for statistical comparison. The statistical tests were two tailed, and a P-value < 0.05 was considered statistically significant.

### Ethics approval and consent to participate

The study protocol complied with the Declaration of Helsinki and was approved by the institutional review board of Hokkaido University Hospital (IRB approval number: 018-0322). The patient provided written informed consent prior to enrollment. The animal study protocol was approved by the Institutional Animal Care and Use Committee (IACUC) of Hokkaido University, and all experiments were performed in accordance with the regulations of IACUC of Hokkaido University (approval numbers 22-0037). All methods are reported in accordance with ARRIVE guidelines (https://arriveguidelines.org) for the reporting of animal experiments under ethics approval and consent to participate.

## Supplementary Information

Supplementary Information.

## Data Availability

The datasets generated and analysed during the current study are available from the following DDBJ Sequence Read Archive (DRA), Accession Number: DRR438367-DRR438369.
