# Supplementary material for: Eribulin inhibits growth of cutaneous squamous cell carcinoma cell lines and a novel patient-derived xenograft
Source: Sci Rep. 2023 May 27;13:8650. doi: 10.1038/s41598-023-35811-3 (PMC10224945; doi:10.1038/s41598-023-35811-3)
Supplement: Supplementary file 1 — Supplementary Information. [file 41598_2023_35811_MOESM1_ESM.pdf]

・Title:

Eribulin inhibits growth of cutaneous squamous cell carcinoma cell lines and a novel patient-derived xenograft

・Authors' names:

Che-Yuan Hsu[1], Teruki Yanagi[1*], Takuya Maeda[1], Hiroshi Nishihara[2], Kodai Miyamoto[1], Shinya Kitamura[1], Keiko Tokuchi[1], Hideyuki Ujiie[1]

Supplemental Table S1. Next-generation sequencing-based multiplex gene assay (PleSSision-160)

ABL1	AKT1	AKT2	ALK	AMER1	APC	AR	ARID1A
ARID2	ASXL1	ATM	ATRX	BAP1	BCL6	BCOR	BRAF
BRCA1	BRCA2	BRIP1	BTK	BUB1B	CARD11	CBL	CBLB
CD79A	CD79B	CDC73	CDH1	CDK12	CDK4	CDKN2A	CHEK2
CIC	CREBBP	CRLF2	CSF1R	CTNNB1	CYLD	DAXX	DDB2
DDR2	DICER1	DNMT3A	ECT2L	EGFR	EP300	EPCAM	ERBB2
ERBB3	ERBB4	ERCC5	ESR1	EZH2	FAM46C	FANCA	FANCD2
FANCE	FAS	FBXO11	FBXW7	FGFR2	FGFR3	FH	FLCN
FLT3	FUBP1	GATA1	GATA2	GATA3	GNA11	GNAQ	GNAS
GPC3	GRIN2A	H3F3A	HIST1H3B	HNF1A	HRAS	HSPH1	IDH1
IDH2	IKZF1	IL6ST	IL7R	JAK1	JAK2	JAK3	KDM6A
KDR	KIT	KLF6	KMT2D	KRAS	MAP2K1	MAP2K2	MAP2K4
MAP3K1	MAP4K3	MDM2	MED12	MEN1	MET	MLH1	MSH2
MSH6	MTOR	MUTYH	MYC	MYD88	NF1	NF2	NFE2L2
NFKBIA	NOTCH1	NOTCH2	NPM1	NRAS	PALB2	PAX5	PBRM1
PDGFRA	PHF6	PIK3CA	PIK3R1	PMS2	PPP2R1A	PRDM1	PRKAR1A
PTCH1	PTEN	PTPN11	RAC1	RB1	RET	ROS1	SDHB
SETD2	SF3B1	SLC7A8	SMAD4	SMARCA4	SMARCB1	SMO	SPOP
SRC	STK11	SUFU	TERT	TNFAIP3	TNFRSF14	TP53	TSC1
TSC2	TSHR	U2AF1	VHL	WT1	XPC	ZNF2	ZRSR2

Supplemental Table S2. Genetic alterations for samples of different origins

Sample origin	Gene and amino acid change (VAF, %)	
Patient's blood	None	
Patient's lymph node	*TP53* R175Pfs*2 (97.2%)	*BRCA1* S1563C (33.6%)
	ARID2 T1167Lfs*6 (58.3%)	*BRCA1* E1562Q (33.4%)
cSCC-PDX (G1)	*TP53* R175Pfs*2 (99.7%)	*ARID1A* S1513N (11.2%)
	ARID2 T1167Lfs*6 (96.3%)	*ARID1A* P1518T (11.2%)
	CDK12 S1306F (34.5%)	*ARID1A* P1521= (11.2%)
	GRIN2A L1377= (10.9%)	*ARID1A* A1522= (11.2%)
	GRIN2A R1372G (10.9%)	*ARID1A* G1525= (11.2%)
	KMT2D S4480P (41.3%)	*GRIN2A* C1382= (11.0%)
	KMT2D S2149T (76.5%)	*GRIN2A* S906N (12.4%)
	KMT2D I2126L (97.7%)	*PRDM1* P804H (12.8%)
	KMT2D P2108S (95.5%)	*EP300* M138I (10.4%)
	KMT2D S1089R (20.2%)	*EP300* T140S (10.4%)
	CDK12 L21S (72.7%)	*NOTCH1* S1409N (65.9%)
	ARID1A I781M (21.4%)	*FGFR2* L92F (45.0%)
	ARID1A T783S (21.3%)	*ALK* Q180H (68.1%)
	ARID1A M785V (21.3%)	*ALK* R179L (68.1%)
	ARID1A S1544P (82.6%)	*ESR1* Q122H (86.4%)
	FAM46C A297S (23.6%)	*GNAS* E676= (25.1%)
	FAM46C E298D (23.6%)	*GNAS* G679D (27.4%)
	ASXL1 Q588R (20.8%)	*GNAS* T683= (27.6%)
	KDM6A I598V (52.5%)	*AKT2* S374R (16.3%)
	KDM6A I724T (71.0%)	*MTOR* G580A (34.9%)
	KDM6A M754V (61.8%)	*FGFR2* L63M (43.3%)
	KDM6A T794P (60.5%)	*FGFR2* V60L (43.3%)
	BCOR R710S (38.3%)	*FGFR2* A52V (43.0%)
	EP300 N452S (29.2%)	*FGFR2* V49A (43.1%)
	EP300 V2273A (50.1%)	*ROS1* T734M (33.8%)
	GATA3 S382A (34.8%)	*ERBB2* M1092V (14.6%)
	TSC1 A529S (52.6%)	*FGFR3* I416V (13.4%)
	TSC1 P513S (53.3%)	*AKT2* S374G (16.1%)
	CBLB T785P (10.8%)	*BRCA1* S1563C (53.8%)
	PTCH1 G1363S (13.6%)	*BRCA1* E1562Q (53.8%)
	PTCH1 A1353T (13.2%)	*BRCA1* R1541T (52.0%)
	KMT2D R1189H (19.6%)	*BRIP1* N666H (16.6%)

VAF: variant allele frequency

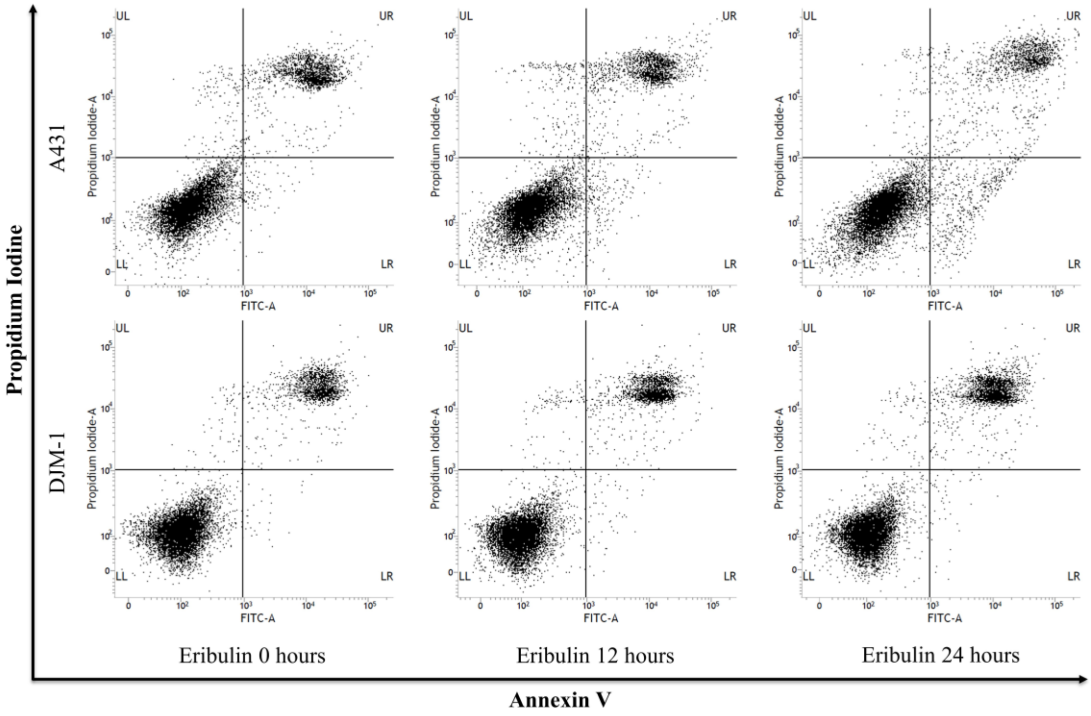

%	Eribulin treatment	Annexin V (−) PI (−) living cell	Annexin V (+) PI (−) early apoptosis	Annexin V (+) PI (+) late apoptosis	Annexin V (−) PI (+) necrosis
A431 cell	0 h	78.32	0.94	19.40	1.34
	12 h	77.53	2.08	18.05	2.34
	24 h	78.36	5.36	15.39	0.89
DJM-1 cell	0 h	82.81	0.31	16.26	0.62
	12 h	77.21	0.59	21.21	0.99
	24 h	74.29	0.45	24.40	0.86

Supplemental Figure S1. Eribulin induces cell death in cSCC cell lines

cSCC cells (A431 and DJM-1) were cultured with or without 0.5 nM of eribulin for 12 and 24 hours. Cells were double-stained with annexin V and propidium iodide (PI), and evaluated by fluorescence-activated cell sorting (FACS) analysis. The x-axis indicates FITC-labeled annexin V fluorescence; the y-axis indicates propidium iodide fluorescence.

Annexin V (−) PI (−) : living cell
Annexin V (+) PI (−) : early apoptosis
Annexin V (+) PI (+) : late apoptosis
Annexin V (−) PI (+) : necrosis
